# Value of baseline characteristics in the risk prediction of atrial fibrillation

**DOI:** 10.3389/fcvm.2023.1068562

**Published:** 2023-02-01

**Authors:** Jiacheng He, Sen Liu, Cuiwei Yang, Yong Wei

**Affiliations:** ^1^Center for Biomedical Engineering, School of Information Science and Technology, Fudan University, Shanghai, China; ^2^Key Laboratory of Medical Imaging Computing and Computer Assisted Intervention of Shanghai, Shanghai, China; ^3^Department of Cardiology, Shanghai General Hospital, Shanghai Jiao Tong University School of Medicine, Shanghai, China

**Keywords:** atrial fibrillation, statistical test, baseline characteristics, risk prediction, electrocardiogram machine

## Abstract

**Introduction:**

Atrial fibrillation (AF) is prone to heart failure and stroke. Early management can effectively reduce the stroke rate and mortality. Current clinical guidelines screen high-risk individuals based solely on age, while this study aims to explore the possibility of other AF risk predictors.

**Methods:**

A total of 18,738 elderly people (aged over 60 years old) in Chinese communities were enrolled in this study. The baseline characteristics were mainly based on the diagnosis results of electrocardiogram (ECG) machine during follow up, accompanied by some auxiliary physical examination basic data. After the analysis of both independent and combined baseline characteristics, AF risk predictors were obtained and prioritized according to the results. Independent characteristics were studied from three aspects: Chi-square test, Mann–Whitney U test and Cox univariate regression analysis. Combined characteristics were studied from two aspects: machine learning models and Cox multivariate regression analysis, and the former was combined with recursive feature elimination method and voting decision.

**Results:**

The resulted optimal combination of risk predictors included age, atrial premature beats, atrial flutter, left ventricular hypertrophy, hypertension and heart disease.

**Conclusion:**

Patients diagnosed by short-time ECG machines with the occurrence of the above events had a higher probability of AF episodes, who are suggested to be included in the focus of long-term ECG monitoring or increased screening density. The incidence of risk predictors in different age ranges of AF patients suggests differences in age-specific patient management. This can help improve the detection rate of AF, standardize the management of patients, and slow down the progression of AF.

## 1. Introduction

Atrial fibrillation (AF) is the most common type of supraventricular arrhythmia in clinical practice. Its hazards include: stroke and thromboembolism (1), heart failure (2), myocardial infarction (3), cognitive decline (4), renal function injury (5), and decreased quality of life (6). The incidence of AF in the general population is about 0.4–1% (7), and increases gradually with age for individuals. This is consistent with the content in the 2020 ESC guidelines (8): “Common AF screening strategies include opportunistic or systematic screening of individuals over a certain age (usually > 65 years) or with other characteristics suggestive of increased stroke risk”. Besides, some studies (9–12) have shown that the prevalence and incidence of AF are relevant to other factors such as gender and regions, which may contribute to the assessment of AF risk.

The risk prediction of AF is conducive to early detection, diagnosis, intervention, and standardized treatment of AF, which can avoid complications and further deterioration of the condition; otherwise, paroxysmal AF at the initial stage will progress to permanent AF (13). To reduce AF related mortality through risk prediction, AF risk factors, not just age, must be identified in order to develop effective and targeted interventions. Since ECG machines with analytical and diagnostic capability are an indispensable monitoring means in the medical field (14), multi-year follow-up studies that combine their diagnosis with some physical examination baseline characteristics will help identify AF predictors in addition to age.

The remainder of the paper is organized as follows. Section 2 describes study organization, inclusion criteria of participants, data collection and AF risk prediction methods. Specifically, a total of 18,738 elderly people (aged over 60 years) were enrolled and followed up for 1–4 years. The assessment of baseline characteristics related to AF risk prediction corresponding to each follow-up was recorded as a single task that included independent and combined risk predictors. The obtained results are summarized in Section 3 and next discussed in Section 4. Finally, Section 5 concludes the paper.

## 2. Materials and methods

### 2.1. Organization and participants

Since July 2015, we have provided annual physical examinations for residents aged 60 years and older at four community health centers (Shihudang, Maogang, Xinbang and Dongjing) in Songjiang District, Shanghai, China. The data inclusion criteria of this study were as follows: (1) aged over 60 years old, (2) registered residents in the four above-mentioned towns, and (3) diagnosed without AF by the resting12-lead ECG obtained during the physical examination in 2015. Those who did not agree to undergo the medical examination funded by the local government were excluded. A total of 18,738 participants were examined from July 2015 to December 2020. This is a prospective cohort study approved by the Ethical Review Board of Shanghai General Hospital, Shanghai Jiao Tong University School of Medicine, Shanghai, China (No. 201508).

### 2.2. Data collection

The data used in this study includes physical examination basic data (age, sex, blood pressure, body mass index (BMI), hypertension, diabetes mellitus and cardiovascular disease history) and diagnosis data of ECG machine (sinus arrhythmia, atrial premature beats (APBs), atrial flutter, ventricular premature beat, left ventricular hypertrophy (LVH), ST segment change, etc.). Specific data list is shown in Supplementary material 1. Hypertension was defined as systolic blood pressure (SBP) ≥ 140 mmHg, diastolic blood pressure (DBP) ≥ 90 mmHg, or current antihypertensive therapy. Diabetes mellitus was defined as a previous diagnosis of diabetes mellitus, treatment with oral hypoglycemic agents or insulin, or having a fasting plasma glucose ≥ 126 mg/dL (7.0 mmol/L) or hemoglobin A1c level ≥ 6.5% (15). Heart disease is defined as a previous diagnosis of heart failure or coronary heart disease.

Before the physical examination and ECG monitoring, the subjects should avoid strenuous exercise, radiation examination and other matters. They are expected to keep the whole body relaxed and keep the supine state during the data measurement. The 12-lead ECG recordings were obtained using a MAC 2000 resting ECG machine (GE Healthcare, Milwaukee, Wisconsin, USA) and analyzed by the Marquette 12SL ECG analysis program. The program can label arrhythmia, measure standard intervals, and perform waveform analysis. The diagnosis of the ECG machine was checked by an experienced clinical physiologist. All ECG abnormalities were confirmed and coded according to the Minnesota ECG Coding Classification System by two cardiologists who were unaware of the clinical data. In addition, the three factors including hypertension, diabetes mellitus and cardiovascular disease history were coded 0-1, with 1 indicating a related history. All subjects were expected to undergo an annual follow-up after enrollment, but some participants failed to continue their appointments.

There were more subjects without AF within a fixed time frame, which was denoted as pre_NAF group. In contrast, the subjects with AF episodes detected were denoted as the pre_AF group, and the data distribution is shown in Table 1. The pre_NAF group made up a large proportion, which was also consistent with the fact that non-AF population accounted for a higher proportion in the overall distribution. The 1*^st^* year in Table 1 meant that the subjects were followed up for just one year, and similarly, the 4*^th^* year indicated that the subjects were followed up for four years. Subjects in pre_NAF group were different from healthy population because they may have other types of heart disease besides AF.

**TABLE 1:** Distribution of subjects’ number and age during follow-up.

	pre_AF group		pre_NAF group	
	**Sample number**	**Age (years old)**	**Sample number**	**Age (years old)**
The 1st year	97	72.47 ± 6.37	808	74.72 ± 7.80
The 2nd year	98	74.79 ± 6.94	1,044	72.53 ± 8.32
The 3rd year	75	72.08 ± 6.68	2,174	69.08 ± 7.69
The 4th year	81	70.96 ± 6.64	14,361	67.95 ± 5.94

### 2.3. Preprocessing

NAN values (966 in total) and outliers need to be removed from the data due to biases introduced during manual data registration and measurement. The interquartile ranges of the boxplot were used to detect outliers in the remaining 17772 cases, and the formulae are as follows:


(1) $$\begin{array}{c} L\,W=Q_{1}-1.5\times \left(Q_{3}-Q_{1}\right) \end{array}$$

(2) $$\begin{array}{c} U\,W=Q_{3}+1.5\times \left(Q_{3}-Q_{1}\right) \end{array}$$

where *Q*_*1*_ and *Q*_*3*_ represent the lower and upper quartiles, respectively, and *LW* and *UW* represent the lower and upper edges of the boxplot, respectively.

Sample points outside this range were judged as outliers. The height of the box reflects the degree of data fluctuation to some extent, and the upper and lower edges represent the maximum and minimum values of the data group. Removal of outliers was operated on only three factors (SBP, DBP and BMI) because all indicators except Age, SBP, DBP and BMI were discrete variables coded 0-1 and it is believed that there was no measurement error in age. Then the total data is standardized with max-min normalization method to eliminate the influence of dimension. The formula is as follows:


(3) $$\begin{array}{c} x_{i,j}^{{}^{\prime }}=\frac{x_{i,j}-\min\,\left(x_{j}\right)}{\max\,\left(x_{j}\right)-\min\,\left(x_{j}\right)} \end{array}$$

where *x*_*i,j*_ and $x_{i,j}^{\prime }$ are data in row *i* and column *j* before and after normalization, respectively. *max*(*x*_*j*_) and *min*(*x*_*j*_) are the maximum and minimum data in column *j*, respectively.

### 2.4. Assessment tasks of AF risk predictors

Four assessment tasks corresponding to the follow-up were constructed for the AF risk predictors from the data of the pre_NAF group and the pre_AF group. Repeated random sampling (20 times) was adopted to minimize the bias caused by unbalanced samples.

#### 2.4.1. Machine learning models

Logistic regression (LR) assigns estimation coefficients to the linear model such that the sum of squared residuals between the observed target and the predicted target of the linear approximation in the dataset is minimized.

Support vector machine (SVM) is often used for bivariate classification of data, and its decision boundary is the maximum margin hyperplane solved for the learning samples, which can be transformed into a convex quadratic programming problem. In this study, radial basis functions (RBF) are used as kernel functions.

Random forest (RF) is composed of a set of tree classifiers {*h*(*x*,Θ_*k*_),*k* = 1,…}, where {Θ_*k*_} is an independent identically distributed random vector generated in conformity with the *k*th tree. Each tree votes on the most popular class corresponding to the input vector *x*, which is essentially an integration of multiple decision trees. In this paper, Gini impurity is chosen as the selection criterion of decision trees, which indicates the probability of a randomly selected sample being misclassified in a subset. Supposing the probability that a node is estimated as a different class at position *t* is *p*(*k*|*t*),*k* = 1,2,…,*Q*, and *Q* is the number of sample types, then the Gini index *G*(*t*) is defined as:


(4) $$\begin{array}{c} G\,\left(t\right)=1-\sum_{k=1}^{Q}p^{2}\,\left(k|t\right) \end{array}$$

After preprocessing, the data sampled from the pre_NAF group and the fixed pre_AF group were subjected to 10-fold cross-validation each time, and there was no data overlap between the training set and the test set in the same run. Finally, the results of multiple machine learning models were averaged after feature selection optimization.

#### 2.4.2. Feature selection (FS)

Recursive feature elimination (RFE) uses the backward selection method to compute the feature subset recursively. The steps of the algorithm are as follows:(1) the initial set is trained and the importance of each feature is obtained by an external estimator; (2) the least important features are removed and the remainder are put into machine learning models again for filtering; (3) the above elimination steps are repeated recursively to receive the optimal feature combination. The importance of features is calculated by different supervised learning estimators. In LR and SVM, it is measured by the absolute value of the feature coefficients, that is, the weight *w* corresponding to the independent variable *x*, while RF uses Gini index to estimate the feature importance.

#### 2.4.3. Statistical test

For numerical variables, the normality test was conducted. If each group met the normality, the t-test was performed for inter-group comparison. Otherwise, the median, minimum and maximum were used for statistical description, and the non-parametric test was used for inter-group comparison. Categorical data were compared between two groups with Chi-Square test. And Mann–Whitney U test was used to determine whether there are statistically significant differences between medians of independent sample groups (pattern of AF and NAF).

Cox proportional-hazards model is a semi-parametric regression model, which can simultaneously study the relationship between multiple risk factors and the occurrence time of events. Cox regression has low requirement to data distribution, while multiple linear regression and logistic regression require data distribution to be approximately normal and binomial, respectively. The arguments of Cox regression model can be continuous numerical variables or discrete categorical variables, and stepwise regression method is used to screen effective characteristics from multiple influencing factors. We calculated the hazard ratio (HR) values, confidence intervals (CI) and p-values using both Cox univariate and multivariate regression models to investigate the effect of baseline characteristics on the prediction of AF and to check disruptive factors. p < 0.05 was considered of statistical significance.

### 2.5. Evaluation

The evaluation criterions of the classification results are accuracy (ACC), sensitivity (SEN), specificity (SPEC), F1 score and positive predictive value (PPV). The F1 score is defined as the harmonic mean of precision and sensitivity. Their formulas are as follows:


(5) $$\begin{array}{c} A\,C\,C=\frac{\mathrm{TP}+\mathrm{TN}}{\mathrm{TP}+\mathrm{TN}+\mathrm{FP}+\mathrm{FN}} \end{array}$$

(6) $$\begin{array}{c} S\,E\,N=\frac{\mathrm{TP}}{\mathrm{TP}+\mathrm{FN}} \end{array}$$

(7) $$\begin{array}{c} S\,P\,E\,C=\frac{\mathrm{TN}}{\mathrm{TN}+\mathrm{FP}} \end{array}$$

(8) $$\begin{array}{c} F\,1\,s\,c\,o\,r\,e=\frac{2\times \mathrm{TP}}{N+\mathrm{TP}-\mathrm{FN}} \end{array}$$

where TP, TN, FP, FN and N stand for true positive, true negative, false positive, false negative and the total number of samples respectively.

## 3. Results

### 3.1. Removal of outliers

**FIGURE 1:**
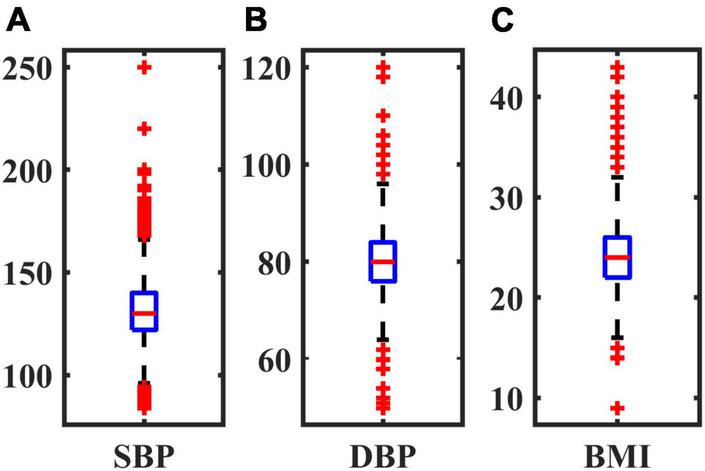
Boxplot of three factors. **(A)** SBP; **(B)** DBP; **(C)** BMI.

The outliers of SBP, DBP and BMI were eliminated respectively. The boxplot in Figure 1 reflects the central location and distribution range of three groups of discrete quantitative data. The number of outliers and the number of remaining samples in the 4 tasks are shown in Table 2.

**TABLE 2:** The number of outliers and number of remaining samples in pre_AF group and pre_NAF group.

	pre_AF group				pre_NAF group			
	**Task1**	**Task2**	**Task3**	**Task4**	**Task1**	**Task2**	**Task3**	**Task4**
SBP outliers	2	0	1	2	15	11	32	200
DBP outliers	1	4	4	1	26	36	65	410
BMI outliers	4	1	2	0	10	8	26	132
NAN	3	7	3	4	61	71	126	691
Remainder	88	86	65	74	699	921	1935	12997

### 3.2. Independent AF risk factors

**FIGURE 2:**
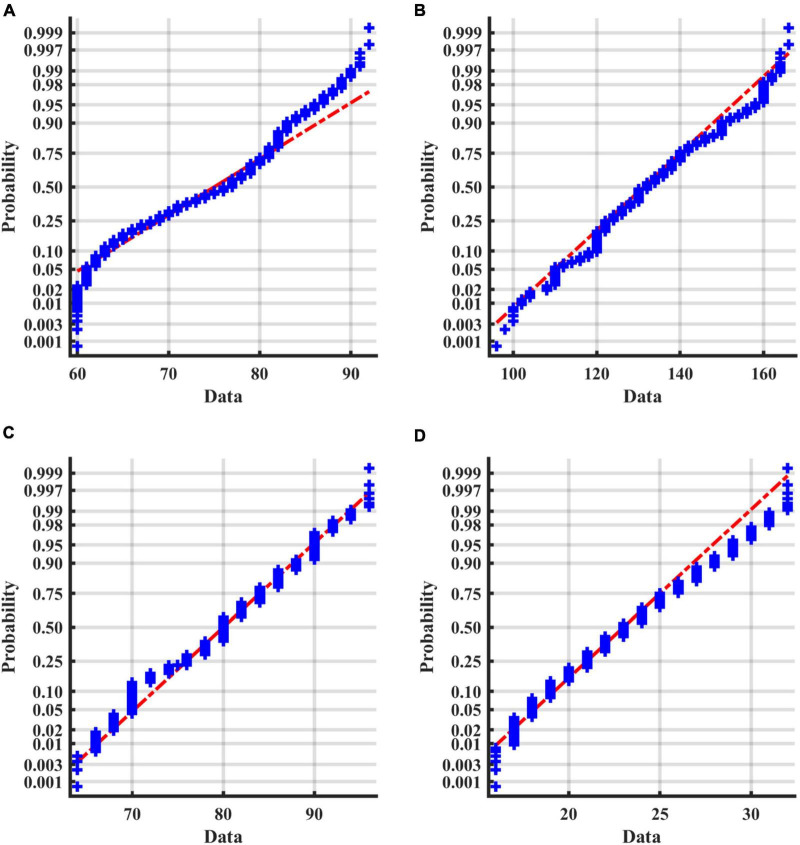
Normal probability diagrams of 4 factors in the pre_NAF group in task1. The probabilities for different values of the factors are shown in blue. If all the sample points are close to the red line, it is reasonable to assume that the samples follow a normal distribution. **(A)** Age; **(B)** SBP; **(C)** DBP; **(D)** BMI.

**FIGURE 3:**
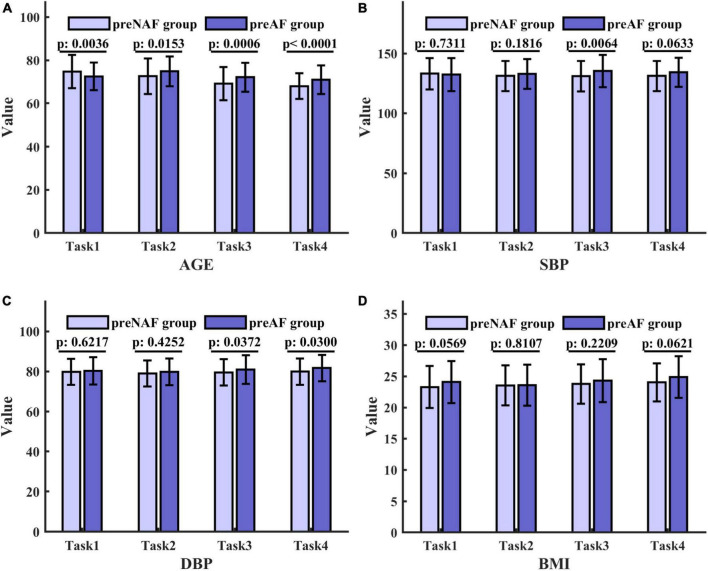
Mean, error bars and *p*-values of 4 numerical variables in the pre_NAF group and pre_AF group in 4 tasks. The height of the columns represents the mean, and the error bar reflects the standard deviation. p values are obtained by the Mann–Whitney U test and indicate the existence of significant differences. **(A)** Age; **(B)** SBP; **(C)** DBP; **(D)** BMI.

For numerical variables (age, SBP, DBP, and BMI), the normality tests were conducted by normal probability diagrams and Figure 2 shows the examples of the pre_NAF group in task1. The non-parametric Mann–Whitney U tests were performed for inter-group comparison on all samples, since two groups of numerical variables in the four tasks cannot meet the normal distribution at the same time. Mean, error bars and p-values of 4 numerical variables shown in Figure 3 indicated that only age was of significant difference in 4 separate tasks between patients who were detected positive for AF and subjects without AF symptoms during the follow-up.

The baseline characteristics of specific subjects for tasks 1 to 4 were shown in Tables 3, 4. Among various categorical variables, only APBs and heart disease were significant under the Chi-square test during the follow-up in at least 3 separate tasks. Due to the small number of subjects recruited in the pre_AF group, the statistical number of indicators coded as 1 tended to be smaller than that in the general elderly population (more than 60 years old).

**TABLE 3:** Subjects’ characteristics at baseline in tasks 1 and 2.

	Task1			Task2		
**Characteristics**	**pre_NAF (*n* = 699)**	**pre_AF (*n* = 88)**	***p*** **-value**	**pre_NAF (*n* = 921)**	**pre_AF (*n* = 86)**	***p*** **-value**
Female gender, *n* (%)	346 (49.5)	42 (47.7)	0.607	517 (56.1)	43 (50.0)	0.199
Age, years old, median	77 (60–92)	72.5 (60–86)	***0.004***	73 (60–93)	74 (60–92)	***0.015***
SBP, mmHg	132 (96–166)	131 (100–166)	0.731	130 (98–166)	132 (106–164)	0.182
DBP, mmHg	80 (64–96)	80 (66–96)	0.622	80 (64–96)	80 (70–96)	0.425
BMI, kg/m^2^	23 (16–32)	24 (16–32)	0.057	23 (16–32)	23 (16–31)	0.811
Sinus bradycardia, n (%)	55 (7.9)	8 (9.1)	0.690	61 (6.6)	8 (9.3)	0.347
Sinus arrhythmia, n (%)	11 (1.6)	6 (6.8)	***0.001***	19 (2.1)	2 (2.3)	0.870
Sinus tachycardia, n (%)	24 (3.4)	2 (2.3)	0.566	30 (3.3)	1 (1.2)	0.282
Atrial premature beats, n (%)	92 (13.2)	18 (20.5)	0.063	77 (8.4)	20 (23.3)	**<*0.001***
Atrial rhythm, n (%)	0 (0)	1 (1.1)	***0.004***	0 (0)	0 (0)	–
Atrial tachycardia, n (%)	2 (0.3)	3 (3.4)	**<*0.001***	5 (0.5)	2 (2.3)	0.057
Atrial flutter, n (%)	2 (0.3)	4 (4.5)	**<*0.001***	0 (0)	0 (0)	–
Junctional premature beat, n (%)	6 (0.9)	3 (3.4)	***0.034***	5 (0.5)	1 (1.2)	0.475
Junctional rhythm, n (%)	0 (0)	1 (1.1)	***0.005***	0 (0)	1 (1.2)	***0.001***
Ventricular premature beat, n (%)	26 (3.7)	4 (4.5)	0.703	35 (3.8)	5 (5.8)	0.361
Short PR interval, n (%)	0 (0)	0 (0)	–	0 (0)	1 (1.2)	***0.001***
First degree atrioventricular block, n (%)	20 (2.9)	0 (0)	0.108	15 (1.6)	2 (2.3)	0.631
Left anterior fascicular block, n (%)	14 (2.0)	0 (0)	0.180	9 (1.0)	1 (1.2)	0.869
Incomplete right bundle branch block, n (%)	14 (2.0)	3 (3.4)	0.392	18 (2.0)	2 (2.3)	0.814
Complete right bundle branch block, n (%)	49 (7.0)	3 (3.4)	0.200	46 (5.0)	3 (3.5)	0.535
Low voltage, n (%)	7 (1.0)	3 (3.4)	0.057	5 (0.5)	1 (1.2)	0.475
Left ventricular hypertrophy, n (%)	124 (17.7)	22 (25.0)	0.098	167 (18.1)	17 (19.8)	0.708
Dilated right atrium, n (%)	1 (0.1)	2 (2.3)	***0.002***	0 (0)	0 (0)	–
ST segment change, n (%)	29 (4.1)	2 (2.3)	0.394	30 (3.3)	4 (4.7)	0.494
T wave abnormality, n (%)	97 (13.9)	8 (9.1)	0.213	100 (10.9)	10 (11.6)	0.827
ST and T change, n (%)	24 (3.4)	5 (5.7)	0.291	35 (3.8)	4 (4.7)	0.696
Hypertension, n (%)	349 (49.9)	46 (52.3)	0.679	431 (46.8)	46 (53.5)	0.235
Diabetes mellitus, n (%)	72 (10.3)	7 (8.0)	0.490	115 (12.5)	2 (2.3)	***0.005***
Heart disease, n (%)	30 (4.3)	16 (18.2)	**<*0.001***	28 (3.0)	8 (9.3)	***0.003***

Bold values represent the *p* < 0.05. Italic values indicate significant differences.

**TABLE 4:** Subjects characteristics at baseline in tasks 3 and 4.

	Task3			Task4		
**Characteristics**	**pre_NAF (n = 1935)**	**pre_AF (n = 65)**	***p*** **-value**	**pre_NAF (n = 12997)**	**pre_AF (n = 74)**	***p*** **-value**
Female gender, n (%)	788 (40.7)	36 (55.4)	***0.018***	7,127 (54.8)	34 (45.9)	0.125
Age, years old, median	68 (60–92)	73 (60–84)	**<*0.001***	67 (60–95)	71.5 (60–87)	**<*0.001***
SBP, mmHg	130 (96–166)	136 (102–160)	***0.006***	130 (96–166)	134 (110–160)	0.063
DBP, mmHg	80 (64–96)	82 (66–94)	***0.037***	80 (64–96)	80 (66–96)	***0.030***
BMI, kg/m2	24 (16–32)	24 (16–32)	0.221	24 (16–32)	24.5 (18–32)	0.062
Sinus bradycardia, n (%)	158 (8.2)	10 (15.4)	***0.039***	1,469 (11.3)	13 (17.6)	0.090
Sinus arrhythmia, n (%)	48 (2.5)	1 (1.5)	0.629	349 (2.7)	2 (2.7)	0.993
Sinus tachycardia, n (%)	39 (2.0)	3 (4.6)	0.151	237 (1.8)	0 (0)	0.241
Atrial premature beats, n (%)	145 (7.5)	13 (20.0)	**<*0.001***	835(6.4)	18 (24.3)	**<*0.001***
Atrial rhythm, n (%)	4 (0.2)	0 (0)	0.714	9 (0.1)	0 (0)	0.821
Atrial tachycardia, n (%)	6 (0.3)	0 (0)	0.653	32 (0.2)	0 (0)	0.669
Atrial flutter, n (%)	3 (0.2)	1 (1.5)	***0.014***	3 (0.0)	0 (0)	0.896
Junctional premature beat, n (%)	9 (0.5)	0 (0)	0.582	52 (0.4)	1 (1.4)	0.199
Junctional rhythm, n (%)	2 (0.1)	0 (0)	0.795	8 (0.1)	0 (0)	0.831
Ventricular premature beat, n (%)	54 (2.8)	5 (7.7)	***0.022***	309 (2.4)	4 (5.4)	0.089
Short PR interval, n (%)	6 (0.3)	0 (0)	0.653	12 (0.1)	0 (0)	0.794
First degree atrioventricular block, n (%)	41 (2.1)	5 (7.7)	***0.003***	235 (1.8)	2 (2.7)	0.565
Left anterior fascicular block, n (%)	19 (1.0)	0 (0)	0.422	129 (1.0)	0 (0)	0.389
Incomplete right bundle branch block, n (%)	16 (0.8)	1 (1.5)	0.539	134 (1.0)	1 (1.4)	0.786
Complete right bundle branch block, n (%)	66 (3.4)	1 (1.5)	0.409	382 (2.9)	0 (0)	0.134
Low voltage, n (%)	14 (0.7)	0 (0)	0.491	68 (0.5)	0 (0)	0.533
Left ventricular hypertrophy, n (%)	316 (16.3)	17 (26.2)	***0.036***	2,237 (17.2)	16 (21.6)	0.317
Dilated right atrium, n (%)	4 (0.2)	0 (0)	0.714	10 (0.1)	0 (0)	0.811
ST segment change, n (%)	67 (3.5)	5 (7.7)	0.072	340 (2.6)	4 (5.4)	0.135
T wave abnormality, n (%)	188 (9.7)	6 (9.2)	0.896	1,260 (9.7)	11 (14.9)	0.134
ST and T change, n (%)	54 (2.8)	1 (1.5)	0.544	251 (1.9)	3 (4.1)	0.187
Hypertension, n (%)	846 (43.7)	45 (69.2)	**<*0.001***	5,680 (43.7)	50 (67.6)	**<*0.001***
Diabetes mellitus, n (%)	175 (9.0)	10 (15.4)	0.083	1,152 (8.9)	6 (8.1)	0.819
Heart disease, n (%)	61 (3.2)	10 (15.4)	**<*0.001***	398(3.1)	4 (5.4)	0.244

Bold values represent the *p* < 0.05. Italic values indicate significant differences.

To investigate whether these baseline variables were appropriate independent AF risk predictors with comprehensive significance over a four-year period, we performed Chi-square test and Mann-Whitney U test for categorical and numerical variables respectively, and Cox regression models were applicable for both variables. Cox regression models required random sampling to avoid unbalanced data distribution. It should be noted that for the inclusion of negative samples, only subjects who successfully completed 4 years of follow-up (12997 negative samples) were considered due to the unstable contact status of the pre_NAF group in the previous 3 years, while all 313 positive samples were included. Considering that some variables appeared more frequently in pre_NAF patients, we excluded them to keep consistent with the objective of this study to find positive AF risk predictors. Table 5 shows baseline characteristics with p-values less than 0.05 under the Chi-square test and Mann-Whitney U test in this case. APBs, LVH and hypertension had an incidence of more than 20% in AF patients. Based on results in Table 5, there were 8 baseline characteristics with HR > 1 and p-value < 0.05 obtained by univariate Cox regression analysis (Table 6), including age, APBs, atrial flutter, junctional premature beat, LVH, ST and T change, hypertension and heart disease. They were identified as independent AF risk predictors finally and supposed to have higher priority in search for independent AF risk predictors.

**TABLE 5:** Baseline characteristics of 12997 negative samples and 313 positive samples with p-values less than 0.05 under the Chi-square test and Mann-Whitney U test.

Characteristics	pre_NAF (n = 12997)	pre_AF (n = 313)	*p*-value
Age, years old, median	67 (60–95)	73 (60–92)	<0.001
SBP, mmHg	130 (96–166)	134 (100–166)	0.002
DBP, mmHg	80 (64–96)	80 (66–96)	0.036
Atrial premature beats, n (%)	835 (6.4)	69 (22.0)	<0.001
Atrial flutter, n (%)	3 (0.0)	5 (1.6)	<0.001
Junctional premature beat, n (%)	52 (0.4)	5 (1.6)	0.001
Ventricular premature beat, n (%)	309 (2.4)	18 (5.8)	<0.001
Left ventricular hypertrophy, n (%)	2237 (17.2)	72 (23.0)	0.008
ST segment change, n (%)	340 (2.6)	15 (4.8)	0.018
ST and T change, n (%)	251 (1.9)	13 (4.2)	0.005
Hypertension, n (%)	5680 (43.7)	187 (59.7)	<0.001
Heart disease, n (%)	398 (3.1)	38 (12.1)	<0.001

**TABLE 6:** Independent predictors for subjects with and without AF (cox proportional hazard model, univariate analysis).

Risk factors list	HR	95% CI	*p*-value
Age	1.038	1.022–1.054	<0.001
Atrial premature beats	1.782	1.364–2.329	<0.001
Atrial flutter	5.093	2.091–12.404	<0.001
Junctional premature beat	2.536	1.047–6.140	0.039
Left ventricular hypertrophy	1.397	1.074–1.818	0.013
ST and T change	1.782	1.023–3.106	0.042
Hypertension	1.260	1.005–1.580	0.045
Heart disease	2.115	1.505–2.972	<0.001

HR, hazard ratio; CI, confidence interval.

### 3.3. Combined AF risk factors

The combined AF risk related baseline characteristics were investigated from two aspects: machine learning models and multivariate Cox regression analysis.

#### 3.3.1. Results of machine learning models

Three typical machine learning models are used to measure predictive ability of all baseline characteristics in tasks 1 to 4 (Table 7). LR models had comparable high predictive performance after parameter optimization in the overall data distribution. Their results were positively correlated with time evolution and the gap between SEN and SPEC narrowed in the last two tasks.

**TABLE 7:** The results of three machine learning models used for all baseline characteristics in 4 tasks.

		ACC (%)	SEN (%)	SPEC (%)	F1 score	PPV (%)
Task1	LR	***60.41***	***55.71***	***64.80***	***0.568***	60.66
	SVM	55.94	46.53	65.21	0.503	57.34
	RF	59.62	50.42	68.59	0.540	***61.23***
Task2	LR	***59.10***	***54.93***	63.21	***0.565***	***60.11***
	SVM	56.76	53.80	59.81	0.547	58.04
	RF	55.78	46.78	***65.03***	0.502	57.54
Task3	LR	66.08	63.69	***68.24***	0.640	***67.38***
	SVM	***66.15***	***71.67***	60.40	***0.675***	66.29
	RF	61.08	55.60	66.10	0.573	62.67
Task4	LR	***65.00***	62.16	***67.87***	***0.635***	***67.78***
	SVM	62.71	***62.64***	62.54	0.622	64.20
	RF	60.20	52.57	68.02	0.563	63.66

Bold and italic values represent the highest value of evaluation criterions in the three classifiers.

By reducing feature dimension and redundancy, RFE method combined with LR models contributed to the establishment of AF risk prediction models (Table 8). The final variable subset was decided by more than half of the votes, named as unified feature selection (unified FS). A slightly lower predictive performance of unified optimal feature collection is acceptable, provided that it had some generalization ability for various optimal sets obtained under different data distributions.

**TABLE 8:** The predictive performance of LR models combined with different FS methods.

	FS	ACC (%)	SEN (%)	SPEC (%)	F1 score	PPV (%)
Task1	Without FS	60.41	55.71	64.80	0.568	60.66
	RFE	65.41	59.93	70.53	0.617	66.35
	RFE and unified FS	64.12	58.50	69.42	0.599	64.61
Task2	Without FS	59.10	54.93	63.21	0.565	60.11
	RFE	61.78	57.79	65.71	0.595	63.22
	RFE and unified FS	61.48	57.22	65.88	0.589	63.45
Task3	Without FS	66.08	63.69	68.24	0.640	67.38
	RFE	67.85	66.43	69.02	0.663	69.13
	RFE and unified FS	66.85	65.36	68.21	0.656	69.28
Task4	Without FS	65.00	62.16	67.87	0.635	67.78
	RFE	67.06	64.25	70.09	0.657	70.35
	RFE and unified FS	65.52	62.40	69.07	0.637	68.64

Table 9 shows important variables selected from the combination of RFE method and unified FS in 4 tasks based on LR models. Variables that appeared more frequently in pre_NAF patients were excluded, even though they were valid for machine learning models. In this case, factors that played a role in all tasks included age, APBs, hypertension and heart disease. Variables valid in three tasks included SBP, DBP, BMI, sinus bradycardia, atrial flutter, junctional premature beat, ventricular premature beat, LVH and ST and T change. There were no variables valid only for tasks 1 or 2. Variables valid only for tasks 3 or 4 included ST segment change. Unstable factors other than the above were ignored due to their seemingly weak association with AF risk prediction.

**TABLE 9:** Important variables in different tasks based on LR models and FS.

Risk factors list	Task1	Task2	Task3	Task4
Gender		✓		✓
**Age**	**✓**	**✓**	**✓**	**✓**
SBP		✓	✓	✓
DBP		✓	✓	✓
BMI	✓		✓	✓
Sinus bradycardia		✓	✓	✓
**Atrial premature beats**	**✓**	**✓**	**✓**	**✓**
Atrial flutter	✓	✓	✓	
Junctional premature beat	✓	✓		✓
Ventricular premature beat		✓	✓	✓
Incomplete right bundle branch block	✓		✓	
Left ventricular hypertrophy	✓		✓	✓
ST segment change			✓	✓
ST and T change		✓	✓	✓
**Hypertension**	**✓**	**✓**	**✓**	**✓**
**Heart disease**	**✓**	**✓**	**✓**	**✓**

#### 3.3.2. Results of multivariate Cox regression analysis

We conducted multivariate Cox regression analysis (Table 10) to measure whether the important independent predictors in Table 6 (univariate Cox regression analysis) were simultaneously positively associated with AF risk prediction and had future practicability. All HR values were more than 1, indicating that they were risk factors and can promote positive outcomes. P-values of variables including age, APBs, atrial flutter, LVH, hypertension and heart disease were less than 0.05, indicating that these independent variables were significant for the interpretation of the whole model. Compared with the four risk predictors obtained by the machine learning models for all tasks, Cox multivariate analysis suggested that LVH and atrial flutter were also important variables. These baseline characteristics are supposed to have higher priority in this cohort study. Besides, we calculated the p-values for the 4 tasks (0.0310, 0.0191, 0.0016, and 0.0015, respectively) by comparing single age factor and combined characteristics using the Mann–Whitney U test. The results indicated a gap between AF risk predictors combination and the single age element suggested by traditional guidelines (8, 16).

**TABLE 10:** Combined predictors for subjects with and without AF (cox proportional hazard model, multivariate analysis).

Risk factors list	HR	95% CI	*p*-value
Age	1.058	1.040–1.075	**<*0.001***
Atrial premature beats	1.494	1.230–1.976	***0.005***
Atrial flutter	3.473	1.355–8.882	***0.009***
Junctional premature beat	1.603	0.660–3.891	0.297
Left ventricular hypertrophy	1.406	1.074–1.841	***0.013***
ST and T change	1.600	0.906–2.823	0.105
Hypertension	1.322	1.051–1.664	***0.017***
Heart disease	1.519	1.051–2.194	***0.026***

HR: hazard ratio; CI: confidence interval. Bold values represent the *p* < 0.05. Italic values indicate significant differences.

### 3.4. Baseline characteristics of subjects at different age ranges

Subjects in the following 7 age ranges were studied separately (Table 11) and it is clear that the incidence of APBs increased substantially with age. The incidence of AF events and heart disease increased in the first six and five intervals, respectively. The probability of LVH increased in the first three ranges, then stabilized at about 21% in people aged 75–89 and only 10.7% in people aged 90 and older. The trend in hypertension was relatively erratic and peaked among participants aged 85–89 years. Similarly, we calculated the incidence of baseline characteristics across age ranges in AF patients alone (Table 12).

**TABLE 11:** The number and proportion of baseline characteristics for all subjects of different age ranges.

Age range (years old)	Sample number	AF (n,%)	APBs (n,%)	LVH (n,%)	Hypertension (n,%)	Heart disease (n,%)
60–64	5465	42, 0.8%	241, 4.4%	812, 14.9%	1958, 35.9%	114, 2.1%
65–69	4719	62, 1.3%	276, 5.8%	765, 16.2%	2084, 44.2%	151, 3.2%
70–74	3279	91, 2.8%	258, 7.9%	610, 18.6%	1719, 52.4%	121, 3.7%
75–79	1985	62, 3.1%	207, 10.4%	427, 21.5%	1007, 50.7%	95, 4.8%
80–84	1204	46, 3.8%	191, 15.9%	262, 21.8%	609, 50.6%	67, 5.6%
85–89	184	9, 4.9%	38, 20.7%	37, 20.1%	103, 56.0%	6, 3.3%
≥90	28	1, 3.6%	7, 25.0%	3, 10.7%	13, 46.4%	1, 3.6%

The proportion was obtained by calculating the ratio of the number of subjects with baseline characteristics to the sample number in the corresponding age range.

**TABLE 12:** The number and proportion of baseline characteristics for subjects with AF of different age ranges.

Age range (years old)	AF patients number	APBs (n,%)	LVH (n,%)	Hypertension (n,%)	Heart disease (n,%)
60–64	42	7, 16.7%	4, 9.5%	20, 47.6%	3, 7.1%
65–69	62	11, 17.7%	14, 22.6%	37, 59.7%	8, 12.9%
70–74	91	16, 17.6%	23, 25.3%	57, 62.6%	13, 14.3%
75–79	62	16, 25.8%	15, 24.2%	38, 61.3%	8, 12.9%
80–84	46	14, 30.4%	11, 23.9%	29, 63.0%	5, 10.9%
85–89	9	5, 55.6%	4, 21.7%	5, 55.6%	0, 0.0%
≥ 90	1	0, 0.0%	1, 100.0%	1, 100.0%	1, 100.0%

The proportion was calculated by considering the probability among AF patients.

## 4. Discussion

AF is the most common supraventricular arrhythmia in clinic. Previous works have reported that approximately 15% to 31% of paroxysmal AF patients at the early-stage progress to persistent or permanent AF during a time period between 4 and 8 years (17). Although AF itself poses little threat to life, ischemic stroke caused by AF is one of the main causes of death (17%) in Chinese community patients with AF (15). To optimize the early management of AF patients and slow down the progression of AF, Chinese guideline (16) provides suggestions for AF risk prediction by age and this has been demonstrated by many studies that there is a positive association between the incidence of AF and advancing age (18–22).

However, AF risk prediction only based on age can fail to screen positive cases and conduct clinical evaluation in a timely and effective manner due to the high proportion of asymptomatic AF patients (23). This study aims to help the progress of AF risk prediction through the diagnosis results of ECG machine that can reflect the changes of cardiac electrophysiology. The principal contributions of this study were as follows: (1) Diagnosis data of ECG machine and some physical examination basic data were explored to create more possibilities for AF risk prediction; (2) Both independent and combined risk predictors of positive correlation with AF were obtained and analyzed in detail, and combined risk predictors are more valuable in clinical applications; (3) This study functions as a preliminary step to reduce the target population for long-term ECG monitoring, which is beneficial to optimize the management of high AF risk population and improve the detection rate of AF.

### 4.1. Advantage of ECG machine for AF risk prediction

As a widely used automated algorithm for computer-based interpretation, GE Healthcare 12SL ECG Analysis Programs used in this study refines itself through regular clinical input and clinically relevant gold standard databases. Although there are small systematic differences between the measurements obtained with automated electrocardiographs from different manufacturers (24, 25), their diagnostic results are similar in differentiating between specific individuals and populations. Nowadays, most of the efficacy tests are skewed to pathologic rhythms with much emphasis on AF (26), which accords with its increasing prevalence and the topic of this study. In the absence of absolute medical definition of waveform fiducial points, the stability of the “gold standard” of human judgment is subject to uncertainty (25). This makes the absolute acceptance of any “gold standard” controversial, even if it is quantifiable. Therefore, the integrated stable analysis algorithms of ECG machines are suitable for clinical applications due to its simplicity, reliability and repeatability. As an extension of analysis algorithms of ECG machines, this study aims to explore effective AF risk predictors in the existing clinical experiment circumstances, and in turn serve the clinical diagnosis. Since the ECG monitoring systems used in this community-based cohort study aims to provide service applied to medical fields instead of non-medical applications (such as sports and elderly activities), performance improvements such as cost, energy efficiency, and battery life are not considered.

It should be pointed out that our focus is on the value of the diagnosis results of ECG machines in the AF risk prediction and there were some limitations in the experimental environment of data acquisition. Therefore, not all AF related factors such as hyperthyroidism were taken into account, but they can be included in the future work.

### 4.2. Evaluation of results

Tables 7, 8 show the average predictive performance of the machine learning models in four tasks. These evaluation indexes basically did not reach 70% but mostly exceed 60%, and there are two possible reasons. On the one hand, this study performed dichotomy tasks between AF and non-AF rather than AF and healthy subjects, and many subjects in the non-AF group actually had other cardiovascular diseases and related complications. The similarity of symptoms may bias the results. On the other hand, the data provided by the ECG machines were the simplified 0-1 code instead of specific values, so some valuable information can be lost in the process. Besides, ACC, SEN and SPEC in task 3 and task 4 were higher than those in task 1 and task 2, indicating that individuals can more possibly have AF attack caused by risk accumulation. Furthermore, some variables may show temporal change, with different distributions in various tasks (e.g., age increases with the task).

### 4.3. AF risk predictors

The independent AF predictors were analyzed by Chi-square test, Mann–Whitney U test and Cox proportional-hazards models. The chi-square test dealt with categorical variables and Mann-Whitney U test dealt with numerical variables, while Cox proportional-hazards models accepted both types of variables. There were 8 baseline characteristics in the intersection of their significance variables sets, including age, APBs, atrial flutter, junctional premature beat, LVH, ST and T change, hypertension and heart disease.

The combined AF risk predictors were determined by Cox regression analysis and LR models with RFE method. The Cox regression models simultaneously assessed the effect of several variables on events, allowing us to examine how specific factors affected the incidence of AF occurring at a given time point. The resulting covariates with HR values greater than 1 and p values less than 0.05 were considered to be significantly positively associated with increased AF risk. The combined baseline characteristics included age, APBs, atrial flutter, LVH, hypertension and heart disease. The p-values (less than 0.05) obtained with the Mann-Whitney U test indicated a gap between combined AF risk predictors and the single age element. Although age is associated with higher AF sensitivity, the consequent sacrifice of specificity may cause anxiety and overdiagnosis. In clinical practice, independent variables cannot fully reflect the outcome variables (positive or negative), and are susceptible to the interference of other variables when they are not completely independent. In particular, AF risk prediction using dozens of variables or a single variable is difficult to implement. Therefore, we focus on the combined AF risk predictors that evolved from univariate analysis.

APBs and AF are arrhythmias of atrial origin. If the large number of APBs indicates atrial fibrosis or electrical activity disorder, it can easily develop into AF in the future. In the community-based Chinese cohort study, we found that the presence of ECG machine-diagnosed APBs was a strong independent and combined predictor of AF risk in the elderly population (≥ 60 years). During the follow-up of 1 to 4 years, the APBs detection rate in this cohort was 22% in patients with AF versus 6.9% in patients without AF. Many studies (27–35) used 24-hour Holters to analyze the relationship between APBs and AF, which can count baseline APBs and use different thresholds to define its frequency of occurrence in the 24-hour recordings. However, the relationship between the count of APBs and the probability of developing AF highly depends on baseline information (such as relevant medical history, medication history, etc.) of the AF-risk population, indicating the limitations of thresholds setting. While in this study, only the occurrence events of APBs reflected by ECG machine with short-term data collection were required to perform AF risk prediction, which could be an effective pre-step before long-term ECG monitoring for high-risk groups. In addition, many strokes occurred without a temporal association with the AF episodes (36, 37), suggesting that frequent APBs may be a stroke risk marker independent of the causal mechanism of AF.

Atrial flutter and AF are proposed to be related entities and may transform into one another (38). Since they usually co-exist before and after medication or ablation, most studies (39–42) have explored the incidence of new AF in patients undergoing ablation. In this study, 16552 patients without AF and 313 patients with AF who had no previous history of successful ablation of atrial flutter were tested by ECG machines. However, the incidence of atrial flutter was only 1.6% in positive samples and only 0.05% in negative samples during the follow-up. This may be affected by the short monitoring period and insufficient follow-up time, and more data is expected to be included.

Left ventricular hypertrophy is usually a compensatory hypertrophy of the heart caused by hypertension. Studies (43–48) investigating the AF predictive role of ECG-based LVH were mainly based the population including both hypertension and normotension. The follow-up period ranged from 3.2 to 11.9 years and the mean age of subjects was 55.4 ± 11 years (49). On this basis, LVH was observed in 258 of 3,235 AF events (8%), compared to 72 of 313 AF events (23%) in the elderly population during the follow-up of 1-4 years in this study. Differences in LVH criteria may limit the confidence of the results and more large randomized controlled studies are necessary. But the differences of results may indicate a higher incidence of LVH in the elderly over 60 years. In addition, there was an apparent correlation between hypertension and LVH in this study, especially for subjects suffering from AF. Among 313 patients with AF, 43 out of 187 (23%) hypertensive patients had LVH and 43 of the 72 patients (60%) with LVH had hypertension. Although LVH was determined to be an independent AF risk predictor, its high occurrence rate in people without AF (2844 cases out of 16552, 17%) may result in inevitable high misdiagnosis rate, as does hypertension (7306 cases out of 16552, 44%). We suggest that LVH and hypertension should be diagnosed in conjunction with other AF risk predictors, rather than independently, in the elderly population.

Heart disease in this study refers to a previous diagnosis of heart failure or coronary heart disease. Recording of histories of heart disease is essential before AF risk prediction in the elderly population, but it is supposed to be combined with other risk predictors for diagnosis to avoid low specificity.

In this study, the positive association between the baseline characteristics above and AF was confirmed in residents aged 60 years and older at four community health centers in China. Their combination is beneficial to reduce the misdiagnosis rate caused by single factor diagnosis for AF risk prediction. Besides, the prioritization of risk predictors can help physicians to specify relevant strategies and help the hierarchical management of AF in specific applications. For patients with a large number of abnormal primary risk factors, the density of AF screening should be strengthened, and long-term monitoring should be performed if necessary. It reflects the nature of this study’s use of short-time ECG recordings as a pre-step to reduce the target population for long-term ECG monitoring. For patients with abnormal range of only secondary risk factors, follow-up, regular physical examination and health education should be carried out to guide patients’ self-health management. For AF patients with relatively stable diagnosis and treatment, routine treatment, rehabilitation and long-term follow-up are expected.

### 4.4. Association of AF risk predictors with age ranges

Given the small sample size of participants over 90, we focused on the first six age ranges. Traditional guidelines consider individuals over 65 years of age to be at high AF risk, and this was confirmed according to the increasing incidence of AF in Table 11. The incidence of APBs was positively correlated with the age range, both for all samples and for AF participants. The incidence of LVH, hypertension and heart disease in all samples increased in the first few age ranges and then stabilized or fluctuated slightly. But in patients with AF, there was a small decline in their incidence in subjects older than 74. Compared with the probability of occurrence in the total sample, the incidence of APBs increased in AF patients (85–89 years old) by up to 34.9% and by an average of 16.5%. The average risk of hypertension in AF patients increased by 9.8%, followed by heart disease (5.9%) and LVH (2.35%). Considering that these baseline characteristics were independent and combined risk predictors, we ranked their importance in terms of their increased probability of AF episodes: APBs, hypertension, heart disease, and LVH. This can provide suggestions for the increase of weights when constructing prediction models with other characteristics that were not covered in this study, or managing AF-related age-specific populations. In addition, APBs was only 1 and 1.4% less common in AF patients and total samples aged 60–64 than in those aged 65–69, respectively (Tables 11, 12). This suggests that the age range for screening high-risk groups can be appropriately extended to over 60 years of age.

## 5. Conclusion

In this community-based cohort study, independent and combined AF risk predictors based on the diagnosis results of ECG machine and some basic physical examination data were explored and analyzed. On the basis of univariate analysis, the recommended combined characteristics included age, APBs, atrial flutter, LVH, hypertension and heart disease, and they were verified superior to the single age factor. The combined AF risk predictors are beneficial to reduce the misdiagnosis rate caused by independent factor diagnosis for AF risk prediction. As a pre-step to reduce the target population for long-term ECG monitoring, the positive association between the baseline characteristics above and AF can provide suggestions for people to be included in the focus of AF screening. In this case, the enhancement of screening density and the arrangement of long-term monitoring for these high-risk population can improve the detection rate of AF, standardize the management of patients, and slow down the progression of AF. Besides, AF risk predictors had different incidence rates in different age ranges, which can provide suggestions for the setting of model weights for the management of AF in specific age groups. Additional data especially in the AF group will be collected in the future, and the study will be expanded to include people aged 30 to 60 years. Furthermore, other influencing factors like Hyperthyroidism will be taken into account.

## Data availability statement

The original contributions presented in this study are included in the article/Supplementary material, further inquiries can be directed to the corresponding authors.

## Ethics statement

The studies involving human participants were reviewed and approved by Ethical Review Board of Shanghai General Hospital, Shanghai Jiao Tong University School of Medicine, Shanghai, China and Songjiang Central Hospital, Shanghai, China. The patients/participants provided their written informed consent to participate in this study.

## Author contributions

JH designed research, performed research, analyzed data, and wrote and edited the manuscript. SL and CY designed research and edited the manuscript. YW designed research, edited the manuscript, and had full access to all of the data in the study. All authors contributed to the article and approved the submitted version.
