# Supplementary material for: Value of baseline characteristics in the risk prediction of atrial fibrillation
Source: Front Cardiovasc Med. 2023 Feb 1;10:1068562. doi: 10.3389/fcvm.2023.1068562 (PMC9928725; doi:10.3389/fcvm.2023.1068562)
Supplement: Supplementary file 1 [file Table_1.DOCX]

**Supplementary material 1.** List of Characteristics.

| Number | Characteristics | Number | Characteristics |
| --- | --- | --- | --- |
| 1 | Gender | 2 | Age |
| 3 | SBP | 4 | DBP |
| 5 | BMI | 6 | Sinus bradycardia |
| 7 | Sinus arrhythmia | 8 | Sinus tachycardia |
| 9 | Sinus pause | 10 | Sinoatrial block (degree II, type I) |
| 11 | Sinoatrial block (degree II, type II) | 12 | Atrial premature beats |
| 13 | Atrial rhythm | 14 | Atrial tachycardia |
| 15 | Atrial flutter | 16 | Junctional premature beat |
| 17 | Junctional escape beat | 18 | Junctional rhythm |
| 19 | Accelerated junctional rhythm | 20 | Junctional tachycardia |
| 21 | Supraventricular tachycardia | 22 | Wandering rhythm |
| 23 | Ventricular premature beat | 24 | Ventricular escape |
| 25 | Ventricular arrhythmia | 26 | Accelerated ventricular rhythm |
| 27 | Ventricular tachycardia | 28 | Short PR interval |
| 29 | First degree atrioventricular block | 30 | Atrioventricular block (degree II, type I) |
| 31 | Atrioventricular block (degree II, type II) | 32 | High-grade atrioventricular block |
| 33 | Third degree atrioventricular block | 34 | Left anterior fascicular block |
| 35 | Left posterior fascicular block | 36 | Incomplete right bundle branch block |
| 37 | Complete right bundle branch block | 38 | Incomplete left bundle branch block |
| 39 | Complete left bundle branch block | 40 | Intraventricular block |
| 41 | Ventricular preexcitation | 42 | Right axis deviation |
| 43 | Left axis deviation | 44 | Low voltage |
| 45 | Poor R-wave progression in the precordial leads | 46 | Left ventricular hypertrophy |
| 47 | Right ventricular hypertrophy | 48 | Dilated left atrium |
| 49 | Dilated right atrium | 50 | Abnormal Q wave |
| 51 | QT prolongation | 52 | ST segment change |
| 53 | T wave abnormality | 54 | Obvious U wave |
| 55 | ST and T change | 56 | Early repolarization |
| 57 | Pacemaker | 58 | Hypertension |
| 59 | Diabetes mellitus | 60 | Heart disease |
